# Decitabine priming prior to low-dose chemotherapy improves patient outcomes in myelodysplastic syndromes-RAEB: a retrospective analysis vs. chemotherapy alone

**DOI:** 10.1007/s00432-016-2331-0

**Published:** 2017-01-20

**Authors:** Li Ye, Yanling Ren, Xinping Zhou, Chen Mei, Liya Ma, Xingnong Ye, Juying Wei, Weilai Xu, Haitao Meng, Wenbin Qian, Wenyuan Mai, Yinjun Lou, Gaixiang Xu, Jiejing Qian, Yejiang Lou, Yingwan Luo, Lili Xie, Peipei Lin, Chao Hu, Jie Jin, Hongyan Tong

**Affiliations:** 1 Department of Hematology, The First Affiliated Hospital, College of Medicine, Zhejiang University, Hangzhou, 310003 Zhejiang China; 2 Institute of Hematology, Zhejiang University, Hangzhou, 310009 Zhejiang China; 3 Myelodysplastic Syndromes Diagnosis and Therapy Center, The First Affiliated Hospital, College of Medicine, Zhejiang University, Hangzhou, 310003 Zhejiang China; 4 Department of Hematology, The Fourth Affiliated Hospital, College of Medicine, Zhejiang University, Yiwu, 322000 Zhejiang China

**Keywords:** Myelodysplastic syndromes, Decitabine, Chemotherapy

## Abstract

**Purpose:**

The aim of this study was to examine whether decitabine priming prior to low-dose chemotherapeutic regimens could improve outcomes in patients with myelodysplastic syndromes—refractory anemia with excess of blasts (MDS-RAEB).

**Methods:**

The current retrospective analysis included all MDS-RAEB patients receiving idarubicin/cytarabine (IA) or aclacinomycin/cytarabine (AA), with or without decitabine priming during a period from February 2010 to May 2015. Treatment response and toxicity were compared between patients receiving decitabine priming and those who did not. A panel of 6 MDS-related genes was examined using bone marrow specimens.

**Results:**

A total of 81 patients were included in the analysis: 40 received decitabine priming prior to chemotherapy (decitabine priming group). The median follow-up was 10.9 months (IQR: 6.2–21.9). The rate of overall response (OR) and complete remission (CR) was significantly higher in the decitabine priming group than in the chemotherapy group (OR: 75.0 vs. 51.2%, *p* = 0.027; CR: 55.0 vs. 29.3%, *p* = 0.019). Overall survival (OS) did not differ significantly between the two groups (19.5 vs. 14.7 months, *p* = 0.082). In a subgroup analysis that included only patients at < 60 years of age, the CR rate in the decitabine priming group was significantly higher than in the chemotherapy group (65.5 vs. 31.0%, *p* = 0.009). Survival benefit of decitabine priming was apparent in patients at < 60 years of age (22.4 months with 95% CI of 6.7–38.1 vs. 14.7 months with 95% CI of 11.4–18.0 months in the chemotherapy group, *p* = 0.028), patients with intermediate and unfavorable karyotypes (22.4 months with 95% CI of 15.1–29.7 vs. 11.9 months with 95% CI of 4.0–19.8 months in the chemotherapy group, *p* = 0.042), and patients with mutated splicing factor genes (35.3 months with 95% CI of 21.4–49.2 vs. 17.8 months with 95% CI of 13.8–21.8 months in the chemotherapy group, *p* = 0.039). Grade 3–4 hematological and non-hematological toxicities were not significantly different between the two groups.

**Conclusions:**

Decitabine priming prior to low-dose chemotherapy could improve treatment responses in patients with MDS-RAEB.

## Introduction

Myelodysplastic syndromes (MDS) are a group of clonal hematopoietic cells disorders characterized by persistent cytopenias and propensity to progression to acute myeloid leukemia (AML) (Ades et al. 2014). According to the International Prognostic Scoring System (IPSS), MDS is classified into low, intermediate-1, intermediate-2 and high-risk groups. Hematopoietic stem cell transplantation (HSCT) is the preferred treatment in intermediate-2- and high-risk MDS patients (Greenberg et al. 2011; Malcovati et al. 2013). For patients not eligible for transplantation, chemotherapeutic regimens similar to that used for AML remains an important approach, with approximately 50% complete remission (CR) rate (Beran et al. 2001; Kantarjian et al. 2007b; Knipp et al. 2007). However, high-intensity chemotherapy is associated with high early stage mortality (around 5–20%) and short survival (6–12 months) in MDS patients (Beran et al. 2001; Kantarjian et al. 2007b; Knipp et al. 2007).

An important advance in the treatment of intermediate- and high-risk MDS is the use of DNA methyltransferase inhibitors. Decitabine (2′-deoxy-5-azacytidine) is a representative demethylating agent that reactivates tumor suppressor genes by demethylating these genes (Kantarjian et al. 2006). In patients receiving decitabine monotherapy, the rate of CR and overall response (OR) has been reported to be 13–39 and 32–70%, respectively (Iastrebner et al. 2010; Kantarjian et al. 2007a, c; Lee et al. 2011; Oki et al. 2012; Steensma et al. 2009). Decitabine in combination with a variety of agents, including histone deacetylase inhibitors, thalidomide, and conventional chemotherapeutics, has been developed to treat intermediate- and high-risk MDS and AML (Blum et al. 2007; Daver et al. 2016; Gao et al. 2015; Garcia-Manero et al. 2006; Geng et al. 2016; Jiang et al. 2015; Kirschbaum et al. 2014; Li et al. 2015; Song et al. 2012; Yang et al. 2005; Zhao et al. 2015). Several studies showed that decitabine in combination with chemotherapy improved the outcomes in patients with relapsed/refractory AML or AML transformed from MDS (MDS/AML) (Leonard et al. 2014; Li et al. 2015; Scandura et al. 2011; Song et al. 2012). Studies using MDS/AML and AML cell lines suggested synergistic effects when decitabine exposure was followed by chemotherapeutic drugs (e.g. idarubicin, daunomycin, clarubicin, homoharringtonine and thalidomide) (Li et al. 2014).

Based on these observations, we adopted a regimen of decitabine priming followed by low-dose idarubicin/cytarabine (IA). Though the preliminary trial suggested promising anti-leukemic effects (Ye et al. 2016), it had limitations with varying diseases (MDS, MDS/AML, and AML with no MDS background) and small sample size. In the current study, we examined whether decitabine priming prior to low-dose chemotherapy is superior to chemotherapy alone for MDS with refractory anemia with excess of blasts (MDS-RAEB). Subgroup analyses were carried out based on patient age, WHO classification, karyotypes and mutation status of six genes related to MDS (DNMT3A, IDH1, IDH2, SF3B1, SRSF2 and U2AF1).

## Patients and methods

### Patients

This study was approved by the Ethics Committee of the First Affiliated Hospital, College of Medicine, Zhejiang University. The study included all patients with MDS-RAEB based on the 2008 WHO classification (Vardiman et al. 2009), receiving low-dose chemotherapy regimen, including IA and aclacinomycin/cytarabine (AA), with or without decitabine priming during a period from February 2010 to May 2015. Cases with one or more of the following conditions were excluded from data analysis: (1) secondary MDS; (2) having previously received chemotherapy or any demethylating agent; (3) severe comorbid cardiac, pulmonary, neurologic, or metabolic diseases; (4) malignant tumors; (5) impaired hepatic (serum total bilirubin level ≥ 2 × upper normal limit) or renal (serum creatinine ≥ 2 × upper normal limit) function prior to treatment.

### Treatment regimens

The IA regimen consisted of intravenous infusion of idarubicin (6–8 mg/m^2^/day, d1-3) and cytarabine (100 mg/m^2^/day, d1-7). The AA regimen consisted of intravenous infusion of aclacinomycin (20 mg/day, d1-4) and cytarabine (10 mg/m^2^, q12h, d1-14). Decitabine was delivered at a dose of 20 mg/m^2^/day via intravenous infusion over 1 h for three consecutive days followed by IA or AA regimen. In the low-dose IA regimen, daily idarubicin dosage was reduced to 3 mg/m^2^/day, and lasted for 4–6 days; daily cytarabine dosage was reduced to 10 mg/m^2^, q12h, and lasted for 14 days. In the low-dose AA regimen, daily aclacinomycin dosage was reduced to 10 mg/day, and lasted for 6–8 days; cytarabine was given at a dose of 10 mg/m^2^, q12h, and lasted for 7–14 days. G-CSF was administered (150 µg twice a day) when neutrophil count was lower than 1 × 10^9^/L, and discontinued when neutrophil count elevated to 2 × 10^9^/L. Treatment cycle was repeated every 4 weeks unless upon myelosuppression. Supportive care, including standard antiemetic, blood transfusion and antimicrobial therapy, were given at the physician’s discretion.

### Follow-up

The last follow-up was conducted on February 2016. The median follow-up was 10.9 months (IQR: 6.2–21.9). The overall survival (OS) was defined as the period from the day of diagnosis to the day of death regardless of the cause or the day of HSCT. Data were censored at the last follow-up.

### Evaluation of treatment response and toxicity

Treatment response was assessed using modified International Working Group (IWG 2006) response criteria (Cheson et al. 2006), and categorized to CR, partial remission (PR), marrow CR (mCR), hematologic improvement (HI), stable disease (SD), and treatment failure. OR included CR, PR, mCR and HI. The extent and duration of severe bone marrow suppression was evaluated using the National Cancer Institute (NCI) Common Terminology Criteria for Adverse Event version 3.0 (CTCAE v3.0) (Trotti et al. 2003). Given the fact that the majority of the patients had pre-treatment neutropenia or thrombocytopenia, we documented duration of grade 3–4 hematologic toxicity in the CR patients during treatment. Grade 3–4 non-hematological toxicities were also evaluated.

### DNA sequencing

Bone marrow mononuclear cells were used to sequence six MDS-related genes, including three epigenetic regulatory genes (DNMT3A, IDH1, IDH2) and three splicing factor genes (SF3B1, SRSF2, and U2AF1). DNA segments that were sequenced were: exon 17/18 of DNMT3A (NM_175629.2) (Ahmad et al. 2014), exon 4 of IDH1 (NM_001282387.1) (Yan et al. 2009), exon 11 of IDH2 (NM_002168.3) (Ahmad et al. 2014), exon 13–16 of SF3B1 (NG_032903.2) (Brecqueville et al. 2012; Rossi et al. 2011), exon 1 of SRSF2 (NM_003016.4) (Patnaik et al. 2013), and exon 2/6 of U2AF1 (NM_001025203.1) (Patnaik et al. 2013).

### Statistical analysis

Statistical analysis was conducted using the SPSS 22.0 software (SPSS Inc.; Chicago, IL, USA). The baseline characteristics and toxicities were compared using the Mann–Whitney *U* test for two independent samples. Categorical variables were analyzed with the Chi-square test or the Fisher’s exact test. Survival curves were constructed by the Kaplan–Meier method and compared by the log-rank test. Statistical significance was set at *p* < 0.05 (2-sided). Factors associated with CR and OS were analyzed using a stepwise approach: first with univariate analysis, followed by multivariate COX or logistic regression if *p* was < 0.10 in the univariate analysis. The factors entered into the initial regression model as independent variables included: sex, age, blood cell count, WHO classification, cytogenetic risk, treatment allocation, and splicing factor and epigenetic regulatory gene mutations.

## Results

### Patient characteristics

A total of 81 patients were included in data analyses. Among them 41 patients received low-dose chemotherapy (*n* = 17 for IA; *n* = 24 for AA), and 40 received decitabine priming prior to chemotherapy (*n* = 23 for IA; *n* = 17 for AA). Patient baseline characteristics, including age, sex, blood cell count, cytogenetic and IPSS risk classifications, were generally comparable between the two groups (Table 1). The percentage of RAEB-2 was not significantly different between the chemotherapy group (65.9%, 27/41) and the decitabine priming group (80%, 32/40) (*p* = 0.152). Mutation status of splicing factor or epigenetic regulatory genes was also comparable (Table 2).

**Table 1:** Baseline characteristics

	Chemotherapy (*n* = 41)	Decitabine priming (*n* = 40)	*p* value
Sex, *n* (%)			0.722
Male	23 (56.1%)	24 (60.0%)	
Female	18 (43.9%)	16 (40.0%)	
Median age			
(IQR; years)	55 (41–61)	55 (39–62)	0.860
Neutrophil count			
(IQR; ×109/L)	1.5 (1.1–2.9)	1.5 (1.1–3.4)	0.709
Hemoglobin level			
(IQR; g/L)	73 (60–86)	75 (61–95)	0.385
Platelet count			
(IQR; ×109/L)	51 (34–85)	52 (33–86)	0.745
WHO classification, *n* (%)			0.152
RAEB-1	14 (34.1%)	8 (20.0%)	
RAEB-2	27 (65.9%)	32 (80.0%)	
Cytogenetic risk group, *n* (%)			0.541
Favorable	23 (56.1%)	28 (70.0%)	
Intermediate	9 (22.0%)	7 (17.5%)	
Unfavorable	5 (12.2%)	2 (5.0%)	
Unknown	4 (9.7%)	3 (7.5%)	
IPSS risk, *n* (%)			0.364
Intermediate-1	9 (22.0%)	7 (17.5%)	
Intermediate-2	18 (43.9%)	25 (62.5%)	
High	10 (24.4%)	5 (12.5%)	
Unknown	4 (9.7%)	3 (7.5%)	

**Table 2:** Gene mutation status

	Chemotherapy (*n* = 41)	Decitabine priming (*n* = 40)	*p* value
Gene mutation status, *n* (%)			
Mutated (≥1 gene)	14/30 (46.7%)	12/25 (48.0%)	0.921
Splicing factor gene mutation status, *n* (%)			
Mutated (≥1 gene)	8/29 (27.6%)	9/27 (33.3%)	0.640
SF3B1 mutation	1/30 (3.3%)	0/26	–
U2AF1 mutation	3/37 (8.1%)	2/31 (6.5%)	–
SRSF2 mutation	4/33 (12.1%)	7/32 (21.9%)	0.294
Epigenetic regulatory gene mutation status, *n* (%)			
Mutated (≥1 gene)	6/37 (16.2%)	8/28 (28.6%)	0.230
IDH1mutation	3/38 (7.9%)	6/31 (19.4%)	0.295
IDH2 mutation	2/38 (5.3%)	2/30 (5.3%)	–
DNMT3A mutation	3/37 (8.1%)	0/29	0.330

### Treatment response

In the overall analysis that included all 81 subjects, the rate of OR and CR was 64.2 and 42.0%, respectively. Patients treated with decitabine priming achieved higher OR (75 vs. 51.2% in the chemotherapy group, *p* = 0.027) and CR (55.0 vs. 29.3%, *p* = 0.019) (Table 3).

**Table 3:** Treatment response

	Chemotherapy (*n* = 41), *n* (%)	Decitabine priming (*n* = 40), *n* (%)	*p* value
OR	21 (51.2%)	30 (75.0%)	0.027
CR	12 (29.3%)	22 (55.0%)	0.019
PR	0	0	–
mCR/HI	9 (22.0%)	8 (20.0%)	0.829
SD	8 (19.5%)	3 (7.5%)	0.115
Failure	12 (29.3%)	7 (17.5%)	0.211

*CR* complete remission, *PR* partial remission, *mCR*/HI marrow complete remission/hematologic improvement, *SD* stable disease, *failure* treatment failure, *OR* overall response (CR + PR + mCR/HI)

In the univariate analysis, CR was associated with age (*p* = 0.018), neutrophil count (*p* = 0.034) and treatment (decitabine priming or not, *p* = 0.019). After adjusting age and neutrophil count, decitabine priming remained to be associated with higher CR (OR: 3.214, 95%CI 1.125–9.183; *p* = 0.029).

### Patient survival

**Fig. 1:**
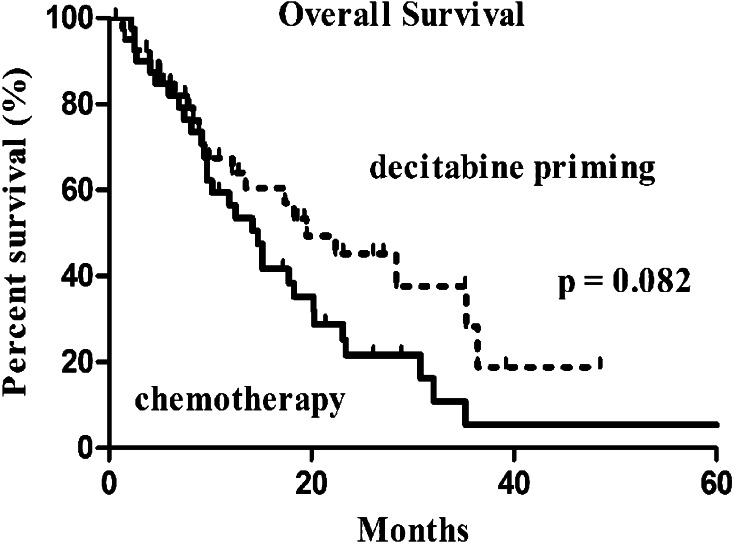
Overall survival in the 2 groups: decitabine priming vs. chemotherapy

**Fig. 2:**
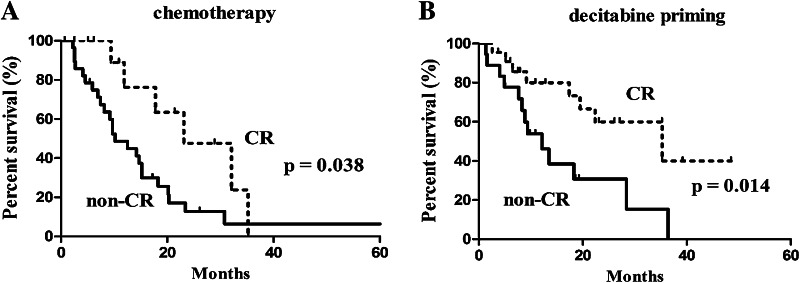
Kaplan–Meier survival analysis in the patients with CR vs. non-CR: **a** chemotherapy, **b** decitabine priming

The median follow-up was 10.9 months (IQR: 6.2–21.9). Of the 81 subjects, six were lost to follow-up (3 cases in each group). OS was not significantly different between the two groups (19.5 months with 95% CI of 9.4–29.6 months in the decitabine priming group vs. 14.7 months with 95% CI of 11.0–18.4 months in the chemotherapy group, *p* = 0.082) (Fig. 1). Patients who achieved CR had prolonged OS regardless of the treatment: 23.1 months (95% CI 9.9–36.3) vs. 10.2 months (95% CI 6.0–14.4) in those not achieving CR (*p* = 0.038) in patients receiving chemotherapy alone (Fig. 2a); 35.5 months (95% CI 12.3–58.3) vs. 12.2 months (95% CI 6.9–17.5) (*p* = 0.014) in patients receiving decitabine priming (Fig. 2b).

In the univariate analysis, OS was associated with sex (*p* = 0.028), cytogenetic risk (*p* = 0.013), treatment (*p* = 0.086) and splicing factor gene mutation status (*p* = 0.089). After adjustment for sex, cytogenetic risk and treatment, mutated splicing factor genes remained to be associated with shorter OS (HR 0.406, 95% CI 0.166–0.990; *p* = 0.048).

### Subgroup analysis

**Fig. 3:**
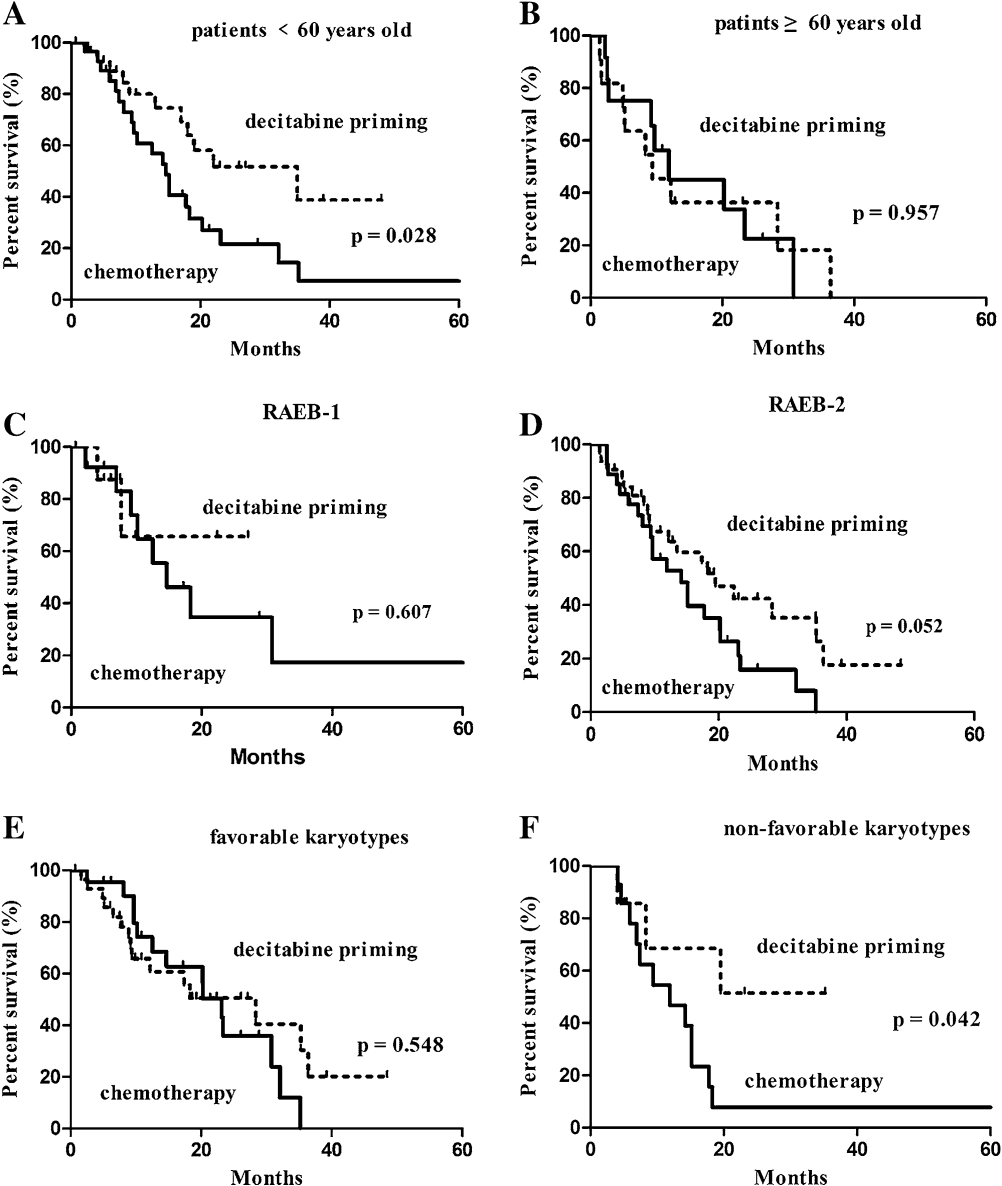
Kaplan–Meier survival analysis: the results of subgroup analysis. **a** Patients <60 years old, **b** patients ≥60 years old, **c** RAEB-1, **d** RAEB-2, **e** favorable karyotypes, **f** non-favorable karyotypes

**Fig. 4:**
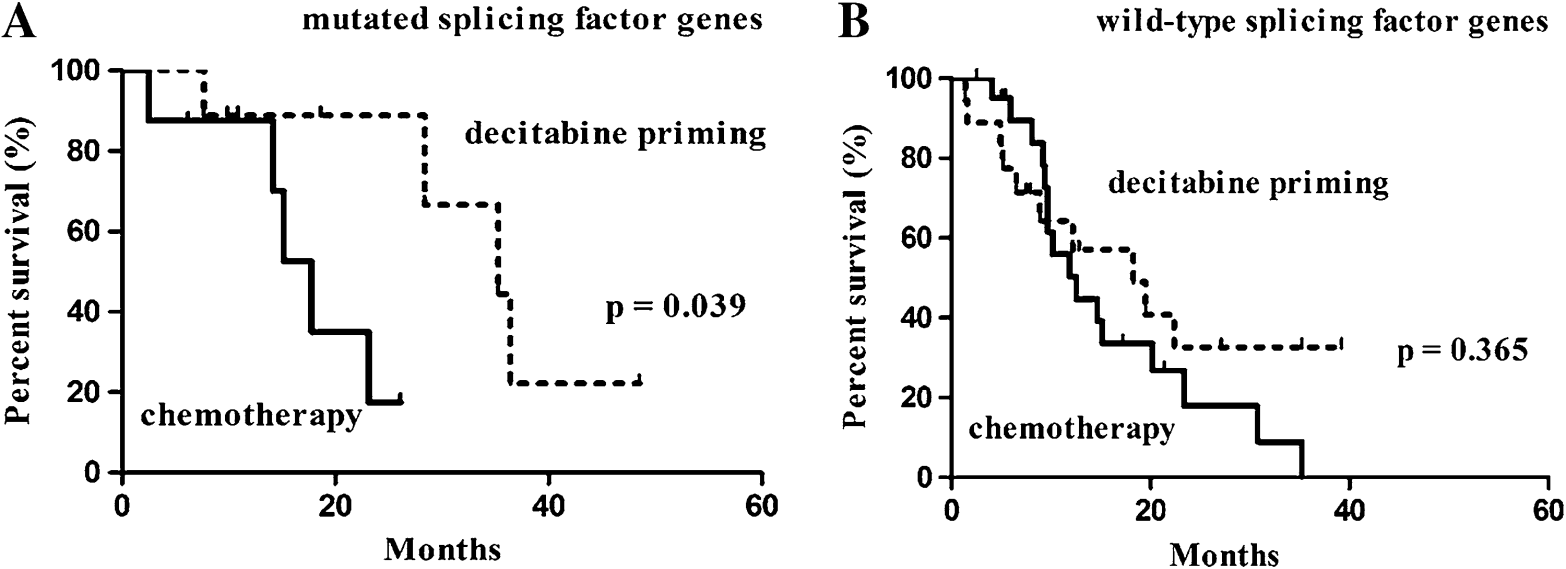
Kaplan–Meier survival analysis: subgroup analysis based on gene mutation: **a** mutated splicing factor genes, **b** wild-type splicing factor genes

A subgroup analysis based on age revealed an association of decitabine priming with higher CR rate (65.5% in the decitabine priming group vs. 31.0% in the chemotherapy group, *p* = 0.009) as well as longer OS (22.4 months with 95% CI of 6.7–38.1 vs. 14.7 months with 95% CI of 11.4–18.0 months, *p* = 0.028) in subjects at <60 years of age (Table 4; Fig. 3a). A Subgroup analysis based on karyotypes revealed an association of decitabine priming with prolonged OS (22.4 months with 95% CI of 15.1–29.7 vs. 9 months with 95% CI of 4.0–19.8 months, *p* = 0.042) (Fig. 3f), but not higher CR (Table 4) in patients with intermediate and unfavorable (non-favorable) karyotypes. A subgroup analysis based on splicing factor genes revealed an association of decitabine priming with prolonged survival (35.3 months with 95% CI of 21.4–49.2 months vs. 17.8 months with 95% CI of 13.8–21.8 months, *p* = 0.039) (Fig. 4a), but not higher CR in patients with mutated splicing factor genes.

**Table 4:** Subgroup analysis

	Chemotherapy (*n* = 41)	Decitabine priming (*n* = 40)	*p* value
Age (years)			
<60	9/29 (31.0%)	19/29 (65.5%)	0.009
≥60	3/12 (25.0%)	3/11 (27.3%)	1.000
WHO classification			
RAEB-1	3/14 (21.4%)	4/8 (50.0%)	0.343
RAEB-2	9/27 (33.3%)	18/32 (56.3%)	0.078
Karyotype			
Favorable	9/23 (39.1%)	15/28 (53.6%)	0.304
Non-favorable	3/14 (21.4%)	6/9 (66.7%)	0.077

Non-favorable karyotypes include intermediate and unfavorable karyotypes

### Toxicities

The rate of grade 3/4 neutropenia (61% in the chemotherapy group vs. 52.5% in the decitabine priming group, *p* = 0.441) and thrombocytopenia (82.9 vs. 75%, *p* = 0.381) was comparable between the two groups. Also, the duration of grade 3/4 neutropenia and thrombocytopenia did not differ significantly between the two groups (Table 5). There was no significant difference in non-hematological toxicities between the two groups (Table 5). One patient in each group died within 4 weeks from the beginning of treatment. The cause of death was cerebral hemorrhage in the chemotherapy group and severe pulmonary infection in the decitabine priming group.

**Table 5:** Toxicities

	Chemotherapy (*n* = 41)	Decitabine priming (*n* = 40)	*p* value
Median duration of neutropenia (IQR; days)	21 (16–35)	22 (13–31)	0.982
Median duration of thrombocytopenia (IQR; days)	26 (20–39)	26 (19–35)	0.752
Grade 3 or 4, *n* (%)			
Infection	28 (68.3%)	28 (70.0%)	0.868
Hemorrhage	13 (31.7%)	11 (27.5%)	0.678
Heart	2 (4.9%)	1 (2.5%)	–
Liver	0	0	–
Kidney	0	0	–
Rash	1 (2.4%)	1 (2.5%)	–

Data for hematological toxicities (neutropenia and thrombocytopenia) were from the patients who had CR after treatment; neutropenia is defined as <1 × 10^9^/L; thrombocytopenia is defined as <50 × 10^9^/L

## Discussion

Potential benefits and risks of decitabine in combination with conventional chemotherapy in patients with myeloid neoplasms have been extensively investigated. In a previous in vitro study with pediatric AML cells, combination of decitabine and cytarabine produced synergistic anti-leukemia effect (Leonard et al. 2014). In a previous study from our research group, decitabine followed by idarubicin produced synergistic anti-leukemia effects in both cultured cells and xenograft animal models (Li et al. 2014). Clinical studies that examined the combination of decitabine and chemotherapeutics, such as standard DA (daunomycin and cytarabine), low-dose AA, and CAG (G-CSF and low-dose AA) suggested CR rate at 50–60% and OR rate at 60–90% in AML and MDS/AML (Li et al. 2015; Scandura et al. 2011; Song et al. 2012). In a previous study (Ye et al. 2016), we reported a CR rate of 43% in MDS, 75% in MDS/AML and 29% in relapsed/refractory AML. The advances in mechanistic and clinical studies (Leonard et al. 2014; Li et al. 2014; Scandura et al. 2011; Song et al. 2012) have led to the use of epigenetic priming in high-risk myeloid neoplasms.

In the current study, we examined whether decitabine priming prior to low-dose chemotherapy (IA or AA) could improve outcomes in intermediate- and high-risk MDS patients. The results revealed increased response rate and prolonged survival in patients treated with decitabine priming prior to low-dose chemotherapy compared with those treated with chemotherapy alone. Consistent with the results of previous clinical trials (Lee et al. 2011; Li et al. 2015; Song et al. 2012), the median OS of patients achieving CR in the current study was significantly longer than that of patients with non-CR regardless of the treatment (decitabine priming or chemotherapy alone). A subgroup analysis in the current study showed a higher CR (65.5%) with a longer OS (22.4 months) in patients at <60 years of age in the decitabine priming group. This finding suggested that patients at <60 years of age could benefit more from decitabine priming treatment. Previous studies suggested that decitabine monotherapy is a better choice for MDS patients with poor karyotypes (Li et al. 2013; Lubbert et al. 2001; Wu et al. 2016). Several studies showed that decitabine in combination with CAG could achieve 50–70% CR in AML and MDS patients with complex karyotypes (Gao et al. 2015; Li et al. 2015). Gao et al. also noted an association of treatment response with the number of courses in AML and MDS patients with complex karyotypes (Gao et al. 2015). Patients with poor karyotypes who received decitabine in combination with CAG tended to have a longer OS (Li et al. 2015). The current study showed longer OS with decitabine priming (22.4 months) in MDS-RAEB patients with non-favorable karyotypes. The CR rate was 66.7% with decitabine priming vs. 21.4% in subjects receiving chemotherapy alone. We believe that such a difference is clinically meaningful despite the lack of statistical significance, presumably due to small sample size.

Mutations of about 40 genes have been identified in MDS. The most frequently mutated genes include SF3B1, U2AF1, SRSF2, ZRSR2, TET2, DNMT3A, EZH2, ASXL1, RUNX1, TP53, STAG2, CBL, and NRAS. Mutated SF3B1 gene is highly enriched in patients with refractory anemia with ringed sideroblasts, and rarely detected in MDS-RAEB patients (Malcovati et al. 2014, 2011; Papaemmanuil et al. 2011). Single SF3B1 mutation has been associated with more favorable prognosis, but may not represent an independent risk factor (Malcovati et al. 2014; Patnaik et al. 2012). Other mutated genes including U2AF1, SRSF2, DNMT3A, IDH1/2, SETBP1 and CBL have been associated with poor survival and progression to AML (Bejar et al. 2011; Graubert et al. 2011, 2012; Haferlach et al. 2014; Kosmider et al. 2010; Makishima et al. 2013; Pardanani et al. 2010; Thol et al. 2012; Walter et al. 2011). In the current study, we examined the mutational status of the three epigenetic regulatory genes (IDH1/2, DNMT3A) and the three splicing factor genes (SF3B1, SRSF2, and U2AF1) in 81 MDS-RAEB patients. The results suggested mutations of splicing factor genes correlated with decreased OS but did not affect the CR. Among patients harboring mutated splicing factor genes, OS was significantly prolonged in the decitabine priming group. These results suggested that patients with mutated splicing factor genes may be suitable for decitabine priming.

AML-type chemotherapy increases early phase mortality (5–20%) and decreases long-term survival (Beran et al. 2001; Kantarjian et al. 2007b). In addition, most of intermediate- and high-risk MDS patients are elderly with diminished function reserve. Based on the above facts, the chemotherapy regimens in the current study were modified (decreased dosage). The low-dose chemotherapy resulted in a lower 4-week mortality (2.5%). Also, grade 3/4 hematological and non-hematological toxicities were tolerated in the current study. These results suggested that decitabine priming did not increase the toxicities of chemotherapy in MDS-RAEB patients.

In summary, the current study suggested that decitabine priming prior to low-dose chemotherapy could improve treatment response and prolong survival in patients with MDS-RAEB. The benefits were most apparent in patients at <60 years of age, with non-favorable karyotypes, and with mutated splicing factor genes. The results of this retrospective study require verification with prospective clinical trials.
